# Mal De Debarquement Syndrome: An Often Unrecognized and Unreported Condition

**DOI:** 10.7759/cureus.64590

**Published:** 2024-07-15

**Authors:** Asif Ali, Sakina Mir, Stephanie Waked, Nylah L Ali, Sami Farooqui

**Affiliations:** 1 Cardiovascular Medicine, UTHealth Houston McGovern Medical School, Houston, USA; 2 Psychology, University of Texas at San Antonio, San Antonio, USA; 3 Internal Medicine, Lebanese American University School of Medicine, Beirut, LBN; 4 English, University of Texas at Austin, Austin, USA; 5 Public Health, University of Texas at Austin, Austin, USA

**Keywords:** vestibular and balance rehabilitation, dizziness vertigo imbalance, vestibular dysfunction, equilibrium, travel-related dizziness, vertigo, mal de debarquement syndrome

## Abstract

Mal de debarquement syndrome (MdDS), also known as "the sickness of disembarkment," is characterized by a persistent bobbing, rocking, or swaying sensation reported by patients long after they have completed travel on a boat or other forms of extended transportation. A detailed patient history, focusing on specific inquiries about recent boat or ship travel, is crucial for a timely diagnosis. The syndrome is unique in that reintroducing similar movements, such as driving, swinging, or returning to the boat, alleviates symptoms temporarily.

We describe the case history of a 28-year-old male who experienced a persistent illusion of ground movement for six months following a fishing expedition. The patient reported alleviated symptoms when re-exposed to movements such as driving or swinging. The patient had undergone extensive medical workups and imaging tests under multiple physicians before being diagnosed with MdDS.

MdDS is a commonly misdiagnosed, underdiagnosed, unreported, and unrecognized condition. Diagnosing MdDS requires a detailed medical and travel history, accompanying an understanding that the symptoms improve upon re-exposure to the same or similar motion.

## Introduction

Mal de debarquement syndrome (MdDS), translating to the "sickness of disembarkment," is characterized by the illusion of movement perceived as an after-effect of prolonged travel on a boat, train, car, or airplane [1-6]. MdDS affects individuals upon disembarkation and is associated with unsteadiness without dizziness. The syndrome occurs when the central nervous system habituates to the rhythmic movement of an extensive trip and subsequently becomes resistant to re-adaptation to stable conditions, which results in a persistent sensation of perceived motion [3,7].

Patients with MdDS commonly describe a sensation of bobbing, rocking, or swaying, often accompanied by headaches, tinnitus, poor attention, chronic fatigue, intolerance to visual motion, anxiety, and panic attacks [1,2,7]. However, rotational vertigo is uncommon [3]. Symptoms can onset immediately after disembarkment or be delayed for one or two days, typically following prolonged movement exposure [2,7]. "Landsickness," or post-motion vertigo upon disembarkment from a cruise or extended travel, usually lasts less than 48 hours [3,8]. While landsickness (sometimes referred to as acute MdDS) often resolves after several hours, in cases of chronic or persistent MdDS, the illusionary movement sensation with subsequent unsteadiness, disequilibrium, and swaying can persist for weeks, months, or even years after the trip [1,4,9].

Factors that can provoke the intensity of MdDS symptoms include lack of sleep, stress from crowds, standing still, and exposure to loud sounds [7,10]. What distinguishes MdDS apart from other forms of sickness, such as motion sickness, airsickness, seasickness, and simulator sickness, is that the reintroduction of specific movements, such as driving, swinging, or motion similar to the initial triggering event, can bring temporary relief to the symptoms [3,8,10,11].

MdDS is believed to be a disorder of neuroplasticity, wherein adaptation to a periodically moving environment alters the functional state of the brain, making it challenging to readapt to stable land conditions [4]. Case series have indicated the absence of inner ear or structural brain abnormalities associated with this syndrome [7]. Neurological and otorhinolaryngology (ENT) tests generally yield unremarkable results, although there have been occasional reports of positional nystagmus [6].

Physical examination, provocative vestibular tests, audiograms, lab results, imaging tests, electroencephalogram (EEG), and electromyogram (EMG) are usually "within normal limits" [2,10]. While these expensive medical tests help exclude other conditions, they do not definitively diagnose MdDS. The key to an accurate diagnosis is to obtain a thorough and accurate patient history, particularly regarding recent trips, especially those involving repetitive motions such as being on a boat [7].

Although no definitive treatment for MdDS exists, some cases may resolve spontaneously [7]. Certain medications, including benzodiazepines, selective serotonin reuptake inhibitors (SSRIs), and serotonin-norepinephrine reuptake inhibitors (SNRIs), have shown potential in alleviating symptoms [1-3,6,7]. Benzodiazepines and amitriptyline may be effective by slowing inappropriate vestibular adaptation [3]. According to Clark and Quick, benzodiazepines may be particularly effective in patients with increased intracortical facilitation mediated by glutamatergic interneurons and N-methyl-D-aspartate receptors [12,13]. Vitamin B­­12 deficiency also reduces symptoms in some MdDS patients [12]. However, reports indicate that meclizine and scopolamine are ineffective in treating MdDS symptoms [7]. Vestibular rehabilitation exercises have been recommended for ongoing symptom management [1,14], while avoiding the motion that triggered the condition is the primary preventive measure once MdDS has resolved [7].

MdDS is a rare condition, often undiagnosed or misdiagnosed, leading patients to seek multiple healthcare providers and undergo numerous unnecessary and expensive tests. Unfortunately, patients are frequently told that their symptoms are psychiatric, causing extreme stress and frustration before receiving an accurate diagnosis. It is important to note that MdDS does not appear to have a psychogenic basis [7]. According to Brown and Baloh, the relatively late onset of symptoms and the absence of other medical complaints are inconsistent with somatization disorder [1].

## Case presentation

A 28-year-old well-developed male presented to the Houston Cardiology Consultants Clinic with chief complaints of dizziness and a persistent swaying sensation over the past six months. These symptoms began after a deep-sea fishing expedition on a small boat, where he experienced no seasickness despite large swells. Post-trip, the swaying sensation continued on land, described as walking on an undulated surface. When the symptoms persisted beyond 24 hours, he sought evaluation at an emergency room but received no diagnosis and was advised to consult his primary care physician. Subsequently, he was referred to an ear, nose, and throat (ENT) specialist, but all tests were negative. With no definitive diagnosis, he underwent further evaluations by other specialists. Over the next six months, his symptoms expanded to include tinnitus, fatigue, anxiety, and depression. Ultimately, a psychiatrist diagnosed him with major depressive disorder and recommended relocation to an apartment 100 miles away for closer care. The psychiatrist then referred him to a cardiologist (AA) for a comprehensive examination.

Upon evaluation, the patient, experiencing anxiety, requested to keep the door open due to claustrophobia and preferred early morning appointments due to developing agoraphobia. Accompanied by his parents, he reported significant life setbacks, including the loss of his girlfriend and job, with discontinued studies due to his undiagnosed condition. The patient and his parents expressed frustration with the numerous unsuccessful consultations and tests. Further questioning revealed symptom relief during specific movements like driving or swinging. Standing still exacerbated his symptoms while viewing complex repetitive patterns on the floor intensified them. Interestingly, riding his motorcycle and spending up to eight hours on a playground swing provided significant relief.

The patient's medical history included anxiety and claustrophobia, with no prior surgeries or history of tobacco, drug, or alcohol use. His family medical history was unremarkable. His vital signs were within normal ranges, including a blood pressure of 120/62 mmHg, a heart rate of 58 beats per minute, and a respiration rate of 14 breaths per minute; he was afebrile. He appeared well-nourished and had an unremarkable physical exam consistent with his age. Despite his distress in the enclosed office setting, he remained alert and oriented, requesting to walk and talk as ambulation provided relief. Motor strength in all muscle groups was preserved, and sensation and deep tendon reflexes were normal. No involuntary movements or cerebellar signs were observed, and his gait appeared normal. During a six-minute walk test, his heart rate increased appropriately, and his oxygen levels remained above 97% with ambulation. Orthostatic tests were negative, and there was no evidence of chronotropic incompetence. Heart sounds at rest and during maneuvers (Valsalva, grip, and squatting) were normal. Other movements, such as the Hall-Pike maneuver, did not affect his symptoms. His finger-stick blood glucose level was unremarkable.

A review of his previous laboratory investigations, including complete blood count with differential and platelets (CBC with D&P), electrolytes, calcium, magnesium, phosphorus, liver function tests, and thyroid tests, was unremarkable. The urine drug test screen showed no abnormalities. His electrocardiogram (ECG) indicated sinus bradycardia, and the exercise stress test demonstrated excellent exercise capacity without any notable findings. The two-dimensional M-mode echocardiogram showed an ejection fraction of 60% with no wall motion or valvular abnormalities. The tilt table test showed no syncope. Chest X-rays displayed clear lung fields and no irregularities. Head CT scan with and without contrast reported no abnormalities or signs of hemorrhaging. MRI and MRA of his head and neck showed no signs of vascular disease or intracranial pathology.

A diagnosis of MdDS was made based on the patient's unique history of symptom resolution during motorcycle riding or prolonged swinging on a playground swing. Negative serologic and imaging markers supported the diagnosis. The patient was prescribed venlafaxine, an SNRI, which effectively alleviated his symptoms. He has consistently followed up with his psychiatrist, and at the time of this publication, he has successfully returned to school.

## Discussion

The history of MdDS follows a stereotypical pattern. Unfortunately, the combination of MdDS symptoms is often misattributed to depression and anxiety, which may coexist with the syndrome but are not the primary cause of the symptoms [7]. The lack of diagnosis and resulting lifestyle changes can significantly contribute to depression and anxiety in patients. Patients struggle to find doctors who believe them and are knowledgeable about this disorder. MdDS presents a challenge for physicians who rely on diagnostic tests to label a patient's symptoms [2]. Multiple healthcare visits are often required before obtaining a diagnosis [11]. Early recognition of the syndrome will allow patients to avoid unnecessary testing [10].

The constant sensation of swaying in MdDS can cause cognitive difficulties, limitations in multitasking, decreased energy, balance problems, and other symptoms. This condition often hinders patients from working or fulfilling their family responsibilities, and some individuals may withdraw from society, as observed in this case.

Most studies on MdDS indicate a higher prevalence among females, typically occurring in the fourth to eighth decades of life [1,3]. Hain et al. identified 27 individuals with MdDS, with 56% experiencing symptom onset between the ages of 40 and 49 and only 11% being younger than 40. In the Hain et al. case report, 80% of the female subjects were either premenopausal or receiving hormone replacement therapy [3]. While precise numbers on the prevalence of MdDS are lacking, a study conducted at the University of California at Los Angeles (UCLA) neuro-otology clinic revealed that 1.3% of patients diagnosed between 2000 and 2005 had MdDS [7].

Macke et al. conducted a study on patients with MdDS, examining their quality of life (QoL) and estimating the economic costs associated with the disorder [11]. The impact of MdDS on patient QoL, including both physical and mental components, was found to be comparable to or even greater than other neurological diseases, such as longstanding multiple sclerosis. Furthermore, the overall economic burden per patient with MdDS appeared to be higher than that of other chronic disorders, such as Parkinson's disease. The study also revealed that, on average, patients with MdDS required 19 healthcare visits before receiving a diagnosis, with approximately one-third of these visits being to primary care physicians. Additionally, patients with MdDS reported undergoing numerous diagnostic imaging procedures, both before and after their diagnosis, highlighting the lack of recognition of MdDS among medical professionals and emphasizing the need for clinical education on this disorder.

## Conclusions

MdDS is widely misdiagnosed, underdiagnosed, unreported, and unrecognized. Our patient presented with various clinical symptoms consistent with MdDS, including the sensation of ground movement after initial motion exposure, swaying, tinnitus, and fatigue. Temporary relief of symptoms was observed with re-exposure to repetitive movements such as swinging. Physical examination and imaging tests showed no significant abnormalities. Furthermore, the hallmark diagnostic indicator of symptom relief with re-exposure to passive motion supported the diagnosis of MdDS. The administration of venlafaxine effectively alleviated many of the patient's symptoms. The key to diagnosing MdDS is to obtain a detailed medical and travel history while recognizing that the symptoms improve upon re-exposure to the same triggering movement or passive motion.
